# Social-ecological dynamics of aggregate mining in Ouagadougou, Burkina Faso

**DOI:** 10.1038/s41598-025-98929-6

**Published:** 2025-04-28

**Authors:** Katharina Salomea Hemmler, Wendinkonté Elisé Gustave Tarpaga, Oussama Himmy, Andreas Buerkert

**Affiliations:** 1 Organic Plant Production and Agroecosystems Research in the Tropics and Subtropics, Universität Kassel, Steinstrasse 19, 37213 Witzenhausen, Germany; 2 Unité de Formation et de Recherche en Sciences de la Vie et de la Terre (UFR-SVT), Université Joseph KI-ZERBO, 03 BP 7021 Ouagadougou 03, Burkina Faso

**Keywords:** Artisanal quarry, Informal sector, Resource extraction, Sand, Urbanization, West Africa, Behavioural ecology, Urban ecology

## Abstract

While traditional materials like clay-based adobe bricks are common in Burkina Faso, rapid urbanization in its capital, Ouagadougou, has driven a shift to more durable construction materials. The city’s demand for aggregates - sand, gravel, and crushed stone - is met by several mechanized and artisanal quarries. To assess the scope and processes of these extraction systems as well as the socio-economic and environmental implications of two artisanal granite quarries (Pissy and Yagma) in Ouagadougou, we employed a mixed-methods approach, including truck counts, aerial imagery for 3D volume estimation, interviews, and air quality measurements. Our findings indicate that sand is predominantly sourced from south of Ouagadougou, with transport distances reaching up to 165 km and an extrapolated annual extraction volume of 1.8 million m^3^, which is relatively low compared to the high urbanization rates and greater volumes extracted in neighboring countries. The 3D model of the Pissy quarry revealed significant terrain alterations and detailed granite extraction data, totaling nearly 450,000 m^3^. Granite quarrying, predominantly carried out by Burkinabe women with limited education, involves cracking, transporting, and crushing rocks. The artisanal quarries displayed a high level of informality, characterized by lack of regulatory compliance, absence of worker contracts and legal protection, manual and small-scale operations, hazardous working conditions, and economic vulnerability and instability. Air pollution varied, with Pissy exhibiting significantly elevated levels of particulate matter. Policy decisions on the future of these quarries should consider health standards as well as the workers’ realities and alternative granite sources.

**Supplementary Information:**

The online version contains supplementary material available at 10.1038/s41598-025-98929-6.

## Introduction

Global urbanization, characterized by rapid rural-urban migration and population growth, is transforming landscapes and economies across the world. Although Africa’s high urbanization rates align with those of other rapidly urbanizing regions globally, the dynamics shaping its urban growth are distinct. Urban expansion in Africa is predominantly characterized by unplanned and unregulated development, compounded by high unemployment rates, with approximately 60% of jobs situated in the informal or grey economy^1^. Additionally, the continent faces challenges such as informal social protection mechanisms and unregulated land markets, alongside inadequate infrastructure and service provision. Governance issues, particularly weak law enforcement by local authorities and ineffective land-use management, contribute to an overall low settlement density. This causes urban sprawl into peri-urban areas on the one side, and very-dense informal settlements on the other^2,3^. Nonetheless, compared with most rural areas, urban dwellers profit from improved economic opportunities and living standards, facilitating enhanced access to essential services such as electricity, piped water, and telecommunication networks^4^. Across the continent, urbanization rates highly vary, ranging from 14.8% in Burundi to 91% in Gabon^5^.

Burkina Faso, despite being among the 15 least urbanized countries on the continent with an urbanization rate of just 32.5% in 2023, exhibits a remarkable urban population growth rate, ranking fourth in Africa at an annual 4.9%^5,6^. Ouagadougou, the capital and cultural, economic, and administrative center of Burkina Faso epitomizes this trend. It is projected to reach a population of 3.5 million by 2025, with an average daily increase of two thousand inhabitants between 2020 and 2025. Ouagadougou is 2.8 times more populous than the country’s second-largest city, Bobo-Dioulasso, marking a significant change from 1950 when both cities had nearly identical populations of approximately 33,000 in Ouagadougou and 36,000 in Bobo-Dioulasso^7^. Recent studies on Burkina Faso’s urban land use and land cover dynamics underscore the rapid urban changes in Ouagadougou compared with Bobo-Dioulasso. In Ouagadougou, there has been a continuous expansion of settlement areas from the inner city towards the periphery, encroaching upon agricultural lands. Data from 2003 to 2021 show a significant shift, with a 78% increase in built-up areas accompanied by a 42% decrease in agricultural land. The most intense changes occurred between 2015 and 2021, highlighting an increase in urban transformations in recent years^8^. In Ouagadougou, general urbanization trends are further enhanced by security concerns in many parts of the country. As the capital has only experienced few terrorist attacks, rural-urban migration is intensified by rising numbers of internally displaced persons (IDPs) searching for refuge in urban centers^9^. As a consequence, there is a growing need for infrastructure, and thus, construction materials. Recent studies on mining-induced vegetation changes have documented an expansion in the total area of quarries surrounding the city over recent decades. Himmy et al.^10^ identified 257 quarry sites, composed of 48% quarries of gravel and 47% of clay, while the remainder were exploited for granite and sand. With increasing urbanization, Ouagadougou’s construction practices are notably shifting from traditional mud/clay blocks (adobe) to concrete structures, viewed as modern and durable^11^. This necessitates a larger supply of construction aggregates such as sand, gravel, and crushed stone. These can be categorized into two types: (1) hard rock aggregates, which are sourced from igneous (granite), sedimentary (sand- / limestone), and metamorphic (marble) rocks through quarrying processes comprising drilling, blasting, and crushing; and (2) sand and gravel aggregates as typically extracted from unconsolidated sediments in rivers, lakes, or oceans^12^. The comprehensive use of aggregates in the construction sector comprises private and commercial buildings, streets, bridges, sewers, and dams^13^.

The extraction of construction aggregates has been associated with diverse environmental and social concerns for both small- and large-scale operations^14^. Internationally, they focus on: (1) the fact that aggregates are non-renewable resources, which poses a risk of depletion for future generations; and (2) the extraction process often causes significant negative environmental impacts, including collateral damage from poor mining practices, transportation inefficiencies, excessive energy consumption, and severe alterations of landscapes^15^. Furthermore, artisanal extraction is characterized as driven by market and poverty^16^, to be non-economic, and labor-intensive^17^. In recent years, sand mining has become a focal point of research due to its associated environmental threats including the erosion of riverbanks^18^, biodiversity reduction^19^, water quality degradation^20^, loss of agricultural land^21^, and as fostering illegal labor practices and the rise of sand mafias^22^. In response to these issues, manufactured sand has emerged as a viable alternative to natural sand, particularly in China, where massive demand is outstripping dwindling sand reserves and exacerbating environmental challenges. This emerging shift towards manufactured sand, which now surpasses natural sources, has been driven by regulatory measures, promotional efforts, and economic factors, leading to diminished environmental concerns^23^.

In this paper, we focus on the construction aggregates crushed stone and manufactured sand from granite, as well as natural sand in and around Ouagadougou, Burkina Faso. Hereby our study aims at determining (i) the scope and processes of various extraction systems, (ii) the socio-economic structures and dynamics at two artisanal granite quarries, and (iii) the effects of artisanal quarrying at these sites on air quality.

## Materials and methods

### Study area

With a total area of 970 km^2^, Ouagadougou is the capital and largest city of Burkina Faso. It lies in the country’s Central Region (12°12′42″ to 12°30′14″ N and 1°41′31″ to 1°21′05″ W, 300 m asl)^8^. The local climate is classified as hot semi-arid (BSh) according to the Köppen-Geiger classification system with a rainy season from April to October and a dry season from November to March^24^.

**Fig. 1:**
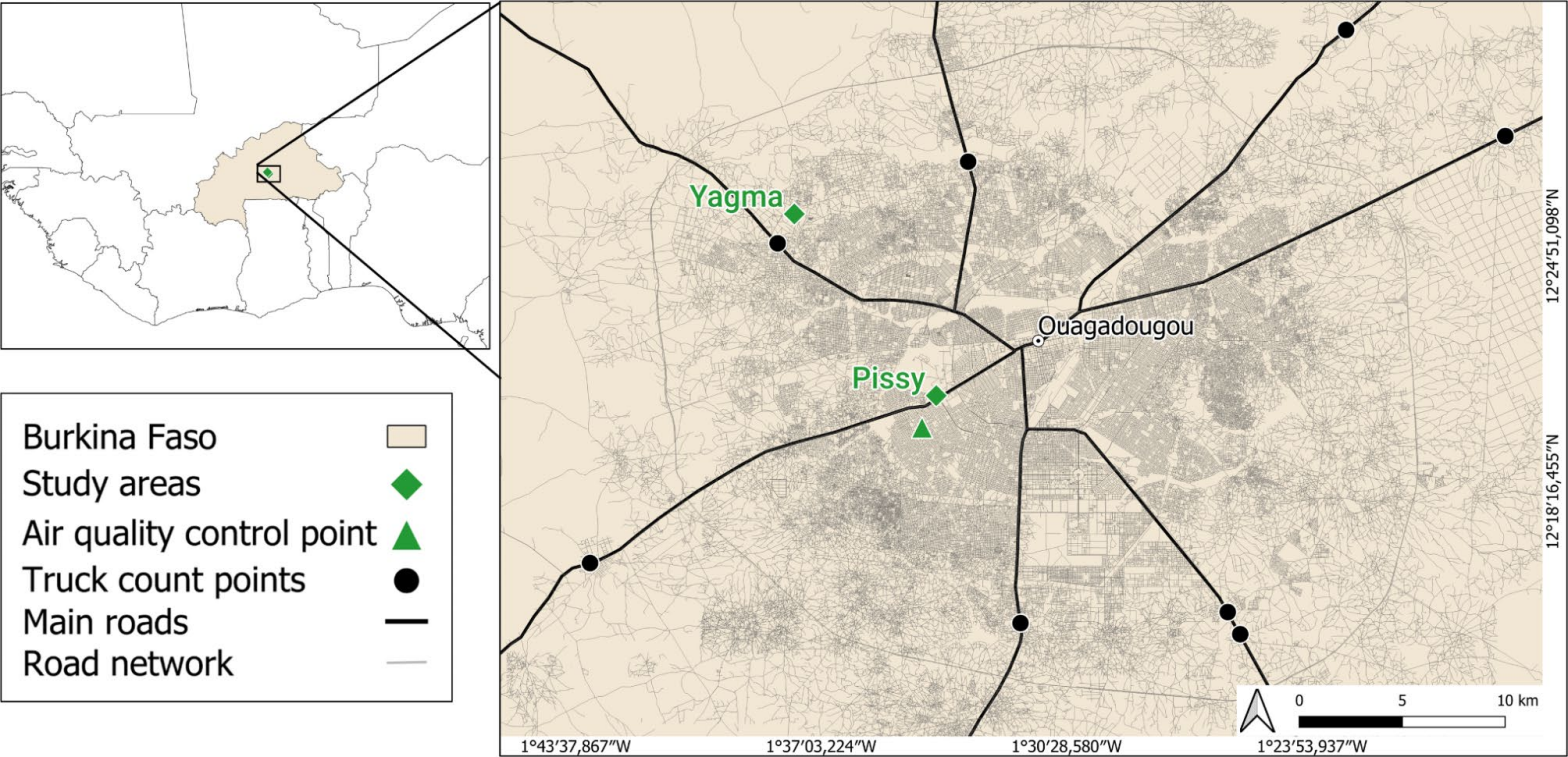
Location of the study area in Ouagadougou, Burkina Faso, West Africa. The figure was generated using QGIS (Quantum Geographic Information System, version 3.16, http://qgis.osgeo.org).

For this study, the granite quarries of Pissy and Yagma were selected due to their artisanal exploitation ongoing for decades, yet providing a distinct contrast in intensity and workforce size (Fig. 1). Pissy, located just 6 km from the city center (12°20’48.3"N 1°33’59.4"W), covers an area of 5.25 hectares, with a length of 350 m, a width of 175 m, and a varying depth, averaging 30 m. Established as an industrial granite quarry in 1990, Pissy transitioned to artisanal extraction after the industrial operations shut down. The working conditions of the predominantly female workforce were characterized by high informality, financial hardship, and safety problems such as the absence of protective gear and lack of social and medical support, which has led to injuries among workers^25,26^. Child labor was also observed^26^, however, over the past decade, lacking childcare was partly alleviated by the construction of a workers’ kindergarten and a primary school. The land is owned by the military of Burkina Faso and surrounded by a military camp and residential areas.

Yagma is situated 16 km away from the city center (12°25’45.6"N 1°37’40.6"W) and encompasses around 7.5 hectares (360 m by 300 m). The site is also known as Alizeta, named after a company that previously operated the granite quarry and still owns the land. Like Pissy, Yagma was originally an industrial granite quarry before becoming an artisanal extraction site in 2012. Yagma has less intensive quarrying activity than Pissy, reflecting the differences in workforce size and extraction volumes between the two locations.

### Data collection and analysis

A mixed methods approach was used for primary data collection to assess the socio-ecological dynamics of aggregate extraction around Ouagadougou. To achieve the first aim of this paper, assessing scope and processes of quarrying activities, we conducted field trips, counts of sand trucks, and used aerial imagery for creating a 3D model. Field trips to sand deposits were undertaken to identify the origin of the aggregates as well as to various granite quarries and sand mines to identify their location and extraction method. Truck counts were conducted on seven major roads leading into the city, with one truck count point on each route, except for National Route 5 (“Manga Road”), which had two measurement points as it was the main route for sand transports (Fig. 1). The counts were carried out over two weeks during August 2022, representing the rainy season, and from the end of May to early June 2024, representing the dry season. A distinction was made between small trucks (6 m^3^ loading volume) and large trucks (28 m^3^) to better capture the volume and scale of sand transportation. The collected data were aggregated to total sums and daily averages, and subsequently extrapolated to estimate the yearly volume of sand transported from surrounding areas into Ouagadougou. Truck count data and calculations can be found in the supplementary material (S1 Data).

In recent years, unmanned Aerial Vehicles (UAVs), or drones, have become essential tools in the extraction industry, particularly for volume estimation. UAVs can provide images of high accuracy with error margins below 3% compared to traditional methods of size recording, while also saving time and allowing frequent monitoring^27–29^. For this study, a DJI Mini 3 Pro was used given its high portability, quality imaging, and reliable performance. The 48-megapixel camera with a 1/1.3” CMOS sensor and an 82-degree field of view provided sufficient resolution for detailed aerial mapping. Individual images of the Pissy quarry were captured at 120 m altitude, with enough overlap to ensure a successful photogrammetric reconstruction. The flight was scheduled mid-morning to take advantage of consistent lighting after pre-flight checks and calibration. Unfortunately, it was not possible to create a 3D model for the Yagma quarry due to insufficient imagery captured, attributed to technical and logistical reasons. The collected 46 UAV images from the Pissy quarry were processed using Pix4D, a photogrammetry software known for its robust capabilities in transforming aerial images into versatile 2D and 3D models. The first step involved inputting the images into the Pix4D desktop software. Here, we carried out image alignment after making necessary adjustments for chromatic aberration, noise reduction, and white balance correction. This process allowed to determine the camera positions corresponding to each picture, as well as the internal orientation parameters of the cameras. Using feature identification and matching techniques a sparse point cloud of the terrain was generated. Subsequently, a dense point cloud was made by densifying the sparse point cloud, which provided a detailed representation of the scene’s geometry. The software generated a 3D textured mesh by triangulating the point cloud and applying photorealistic textures from the original images. A Digital Surface Model (DSM) was interpolated from the point cloud, serving as a base for orthorectification. The volume of extracted material was estimated by comparing pre- and post-extraction DSMs using the DSM differencing technique in Pix4Dmapper. This method allowed the best-possible determination of the material removed based on elevation differences.

For the second goal, determining the socio-economic structures of two granite quarries, a structured questionnaire was conducted with 123 workers in Yagma (*n* = 67) and Pissy (*n* = 56). The higher number of respondents in Yagma was due to the more diversified mining modes at this site, which included workers involved in sand extraction. Within the quarries, workers were selected randomly, and due to low literacy rates, informed consent was obtained verbally for both study participation and the publication of identifying information and/or imagery. All participants were provided with a standardized information script, and their verbal consent was documented electronically by the translator prior to being surveyed. Although children were present and working at the sites, we limited our data collection to adults because the extensive nature of our questionnaire required responses that were likely too complex for children, and due to ethical concerns to ensure special protection for minors in research activities. Prior to the survey, which took place between August 2022 and January 2023, the entire study, including all methods performed in accordance with the relevant guidelines and regulations, was approved by the central ethics committee of the University of Kassel, Germany. The data were collected using the open-source software KoboCollect (version 2.021.47, KoboToolbox, 2022) and translated from the local language Mooré to French. The questionnaire covered sociodemographic data, the scope and conditions of work, cost-revenue structures, regulations, socio-environmental effects, and workers’ perspectives. The survey data were analyzed using descriptive statistics, Pearson’s Chi Square tests, T-Tests, Fisher-Tests, and ANOVA, to understand relationships within the data. The full questionnaire and responses can be found in the supplementary material (S2 Data). Open interviews were conducted with a diverse group of stakeholders, including managers of sand and gravel deposits (*n* = 11), artisanal sand miners (6), sand and gravel truck drivers (5), representatives of an industrial granite quarry (1), and the General Directorate of Quarries (Direction Générale des Carrières, DGC, 1).

**Fig. 2:**
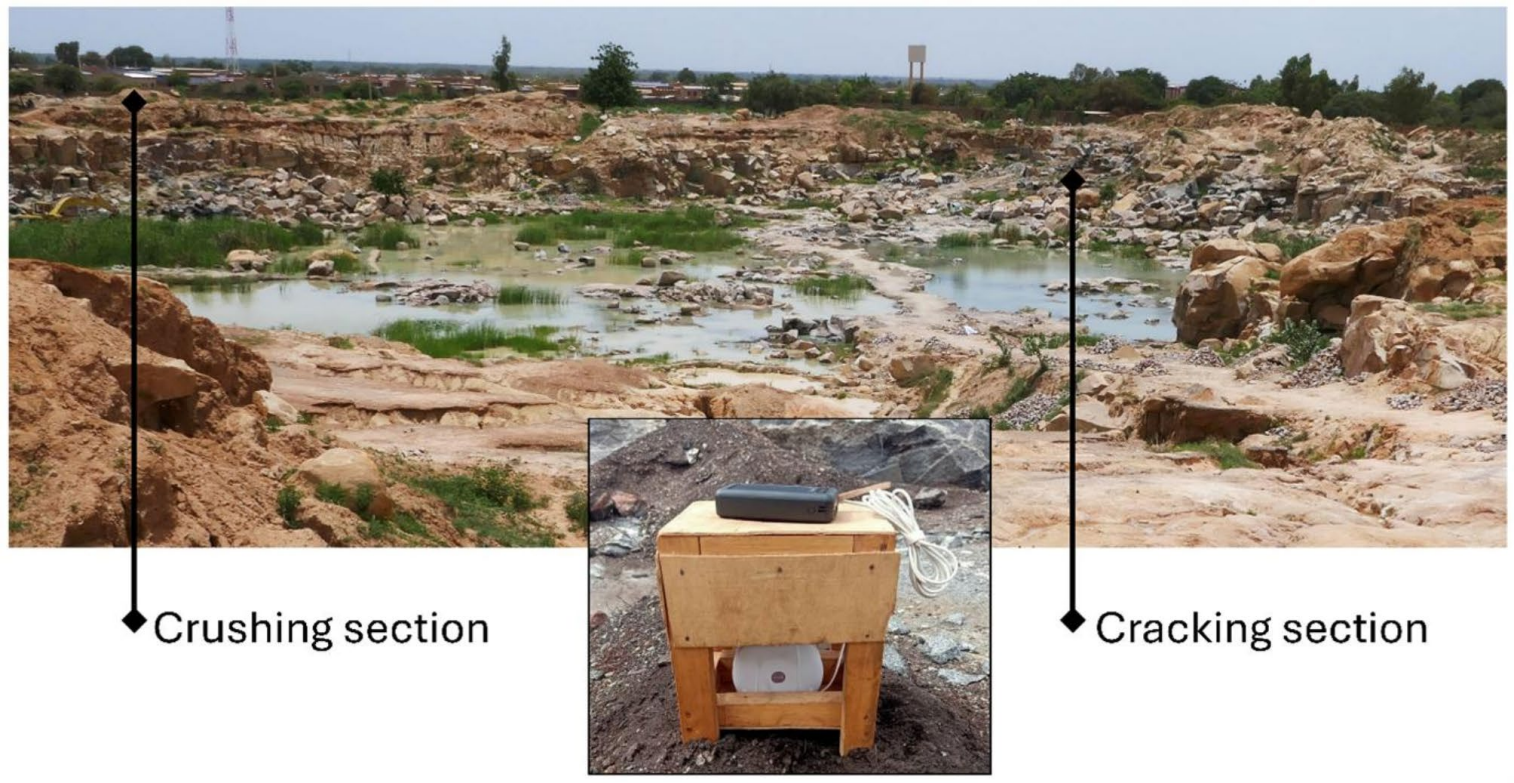
Installation of air quality measurement device (airQ Science, Corant GmbH, Leipzig, Germany) in Yagma, Ouagadougou, Burkina Faso (August 2022).

To assess the effects of artisanal extraction on air quality, measurements were conducted using an air-Q device - Science option (Corant GmbH, Leipzig, Germany) in Pissy and Yagma, at the respective quarry’s lower part (further referred to as “cracking section”) and the upper part (“crushing section”) as well as at a neutral control point within the city (Figs. 1 and 2). This resulted in five measurement days per season, adding up to ten days of data collection in total. The first round of measurements occurred in September 2023 during the rainy season, followed by a second round at the end of October to the beginning of November 2023, during the dry season. Daytime measurements were taken from 8:30 am to 5:30 pm at two-minute intervals. The device was set up in a shaded wood box and powered by a solar power bank (Fig. 2) to ensure continuous operation throughout the measurement periods.

The air quality measurements in this study comprised the determination of concentrations of carbon monoxide (CO), particulate matter (PM_1_, PM_2.5_, PM_10_), nitrogen dioxide (NO₂), sulfur dioxide (SO₂), ozone (O₃), volatile organic compounds (VOCs), temperature, noise, maximum noise (the highest noise level within a two-minute interval), and health and performance indices. These elements were selected due to their known impact on human health and environmental quality. Elevated levels of CO, NO_2_, SO_2_, PM, and ozone are associated with respiratory and cardiovascular diseases^30^. Temperature and sound were also critical factors, as extreme conditions may negatively affect health and work performance. The Health and Performance Indices, proposed by airQ, integrate some of these elements to provide a comprehensive assessment of their effect on human health and well-being. Oxygen, absolute and relative humidity, CO_2_, air pressure, and dew point were also measured by the airQ device but not considered in the analysis. Air quality data can be found in the supplementary material (S3 Data). Data analysis was performed using descriptive statistics and non-parametric tests, specifically the Kruskal-Wallis Test in the R 4.1.2 software, RStudio, and the Tidyverse package^31^. Given the small sample sizes and potential non-normality of the data, the Kruskal-Wallis Test was chosen for its suitability in analyzing the study’s one-day-per-location data structure. Significant findings were analyzed using the Dunn’s Post Hoc test to assess differences among locations.

## Results

### Aggregate extraction around Ouagadougou

**Fig. 3:**
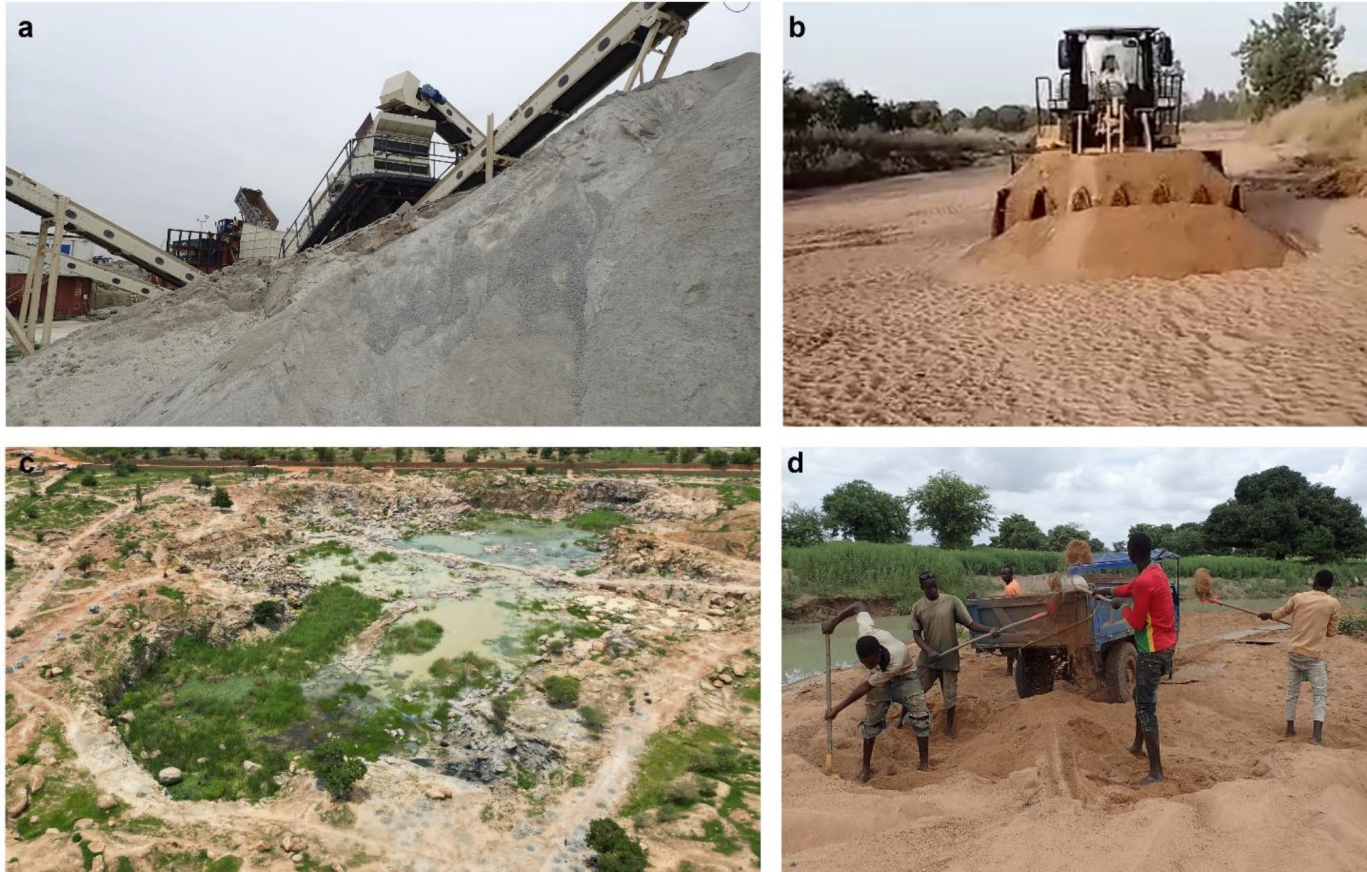
Aggregate mining around Ouagadougou, Burkina Faso (August 2022): (**a**) Industrial granite quarry (Carrière Globex). (**b**) Artisanal granite quarry in Yagma. (**c**) Mechanized sand mining in Bousouma. (**d**) Artisanal sand mining in Gogo.

**Fig. 4:**
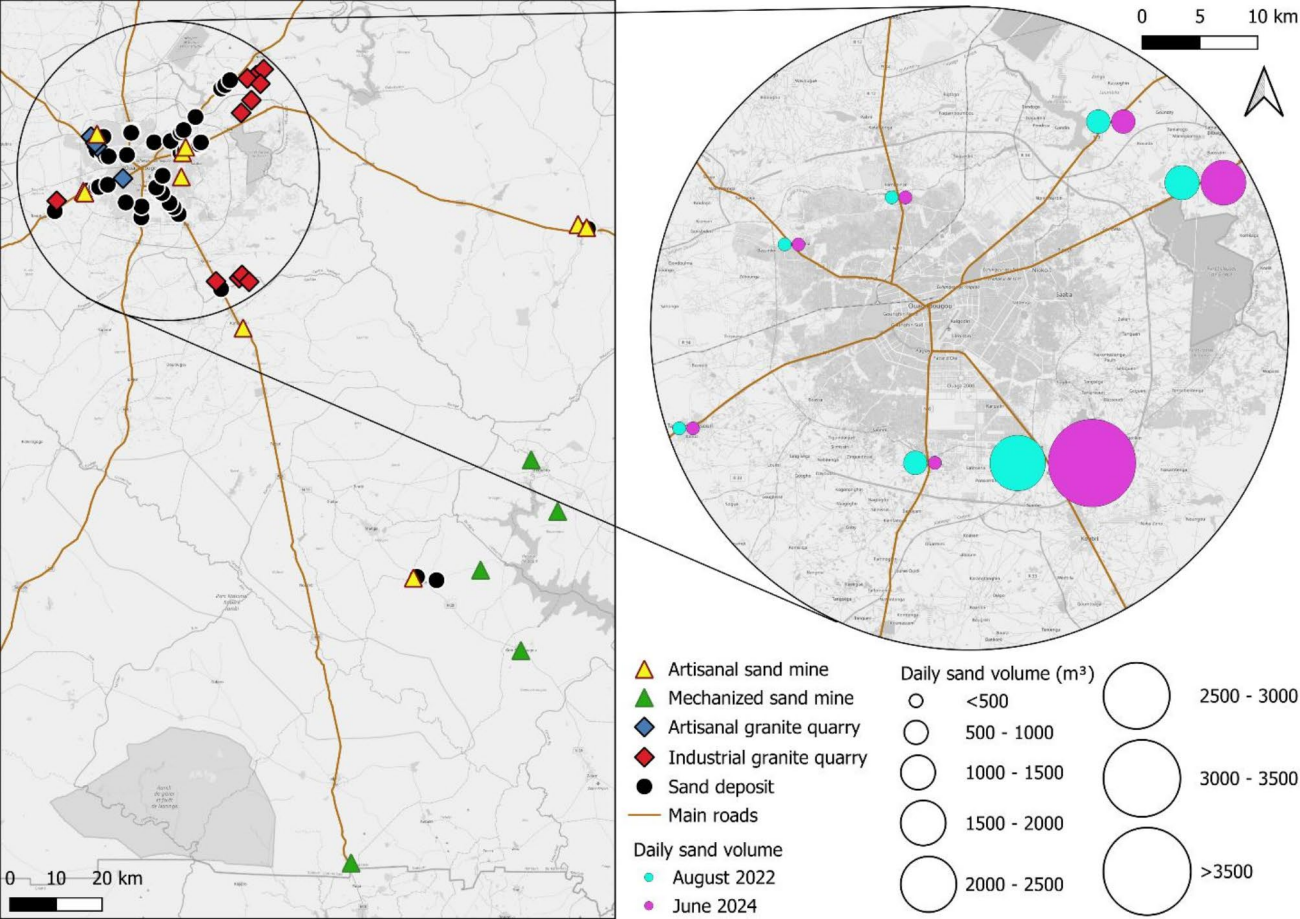
Location of sand deposits, artisanal and mechanized sand mines, artisanal and industrial granite quarries (**a**), daily average volumes of mined sand in August 2022 and June 2024 (**b**) around Ouagadougou, Burkina Faso. The figure was generated using QGIS (Quantum Geographic Information System, version 3.16, http://qgis.osgeo.org) and OpenStreetMap (available under the Open Database License, https://www.openstreetmap.org/copyright).

Several mechanized and artisanal quarries contribute to the supply of construction aggregates for the rapidly growing capital city of Ouagadougou. Industrial quarries, located within a radius of 35 km from the city center, as well as artisanal granite quarries produce crushed stone and manufactured sand, while natural sand originates from intermittent streams and riverbeds at various locations (Figs. 3 and 4). The artisanal sand mines were situated closer to the city center and had relatively low extraction volumes, whereas the mechanized systems extended up to 165 km from Ouagadougou, accounting for the majority of sand production.

The conducted truck counts indicated that the majority of natural sand originated from the capital’s southern and eastern outskirts, while only little quantities were transported from the western and northern regions into town (Fig. 4). National Route 5 alone accounted for 40% (rainy season) and 52% (dry season) of the total truck-based sand transports. The truck counts further revealed that the mined volumes were around 44% higher in the dry season (June 2024) than in the rainy season (August 2022), with total average daily volumes of 7223 m^3^ and 5025 m^3^. Extrapolated throughout the seasons, the yearly mined quantity of natural sand reached around 1.8 million m^3^ or 3.3 Mt, assuming a sand density of 1.85 g/cm^3^.

**Fig. 5:**
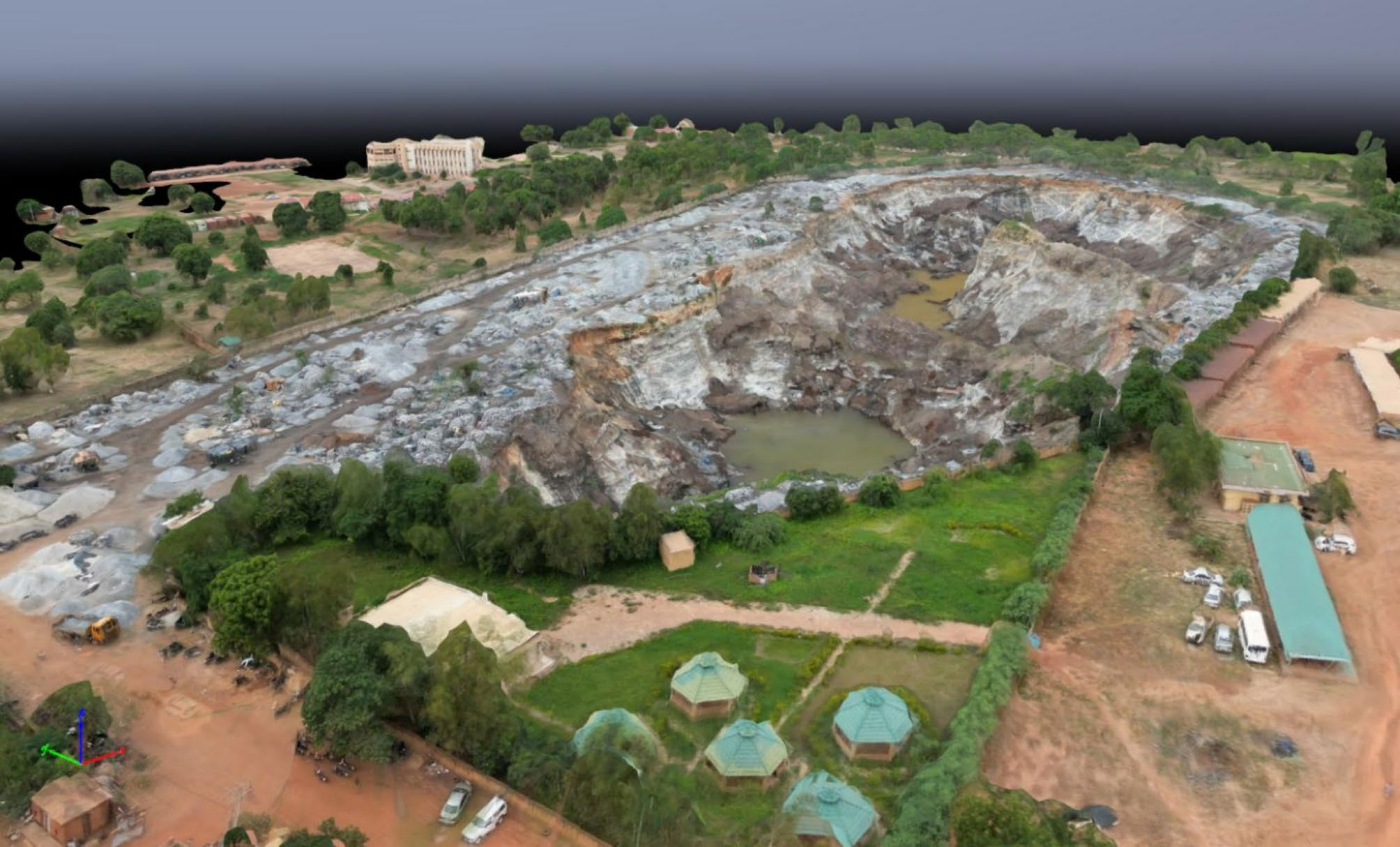
Illustration of a textured 3D model of the artisanal Pissy Quarry, Ouagadougou, Burkina Faso.

The 3D model of the artisanal granite quarry of Pissy (Fig. 5) elucidated detailed terrain alterations and intricate geomorphological features, including slopes, benches, and voids. Comparing the pre-extraction DSM (baseline) with the post-extraction DSM (current terrain) yielded an estimated granite extraction of 447,438 m^3^.

### Operation of the Pissy and Yagma quarries

The sociodemographic analysis of the workers from the granite quarries of Pissy and Yagma revealed that nearly 80% of them were women, with an average age of 40.5 years (18 to 89 years). The educational background showed significant disparities between genders and across different age groups. Men reported an average of 2.9 years of schooling compared with 1.1 years for women (T-Test: *p* = 0.017), and younger respondents (20–30 years) averagely schooled for 4.1 years, while those aged 50 or above reported no formal education at all (ANOVA: *p* = 1.83e-05). 80% of the respondents had no formal education, 10% had completed primary education, 8% had secondary education, and a mere 2% had tertiary education, with significant differences between genders (Fisher Test: *p* = 0.0074). Most respondents were married (72%), while 19% were unmarried and 9% were widowed, and on average, they had 4.3 children. The vast majority of interviewees were Burkinabe (99%), with the remaining coming from Côte d’Ivoire. Respondents had been working at the study sites for an average of 8.2 years, with a significant difference between the two locations (T-Test: *p* < 2.2e-16). Workers in Pissy had typically been active at the site for 15 years, while those in Yagma had been working there for an average of 2.6 years. More than half (55%) of the respondents reported having worked in other sectors such as agriculture, commerce, construction, fashion, or various other industries before joining the quarry.

**Fig. 6:**
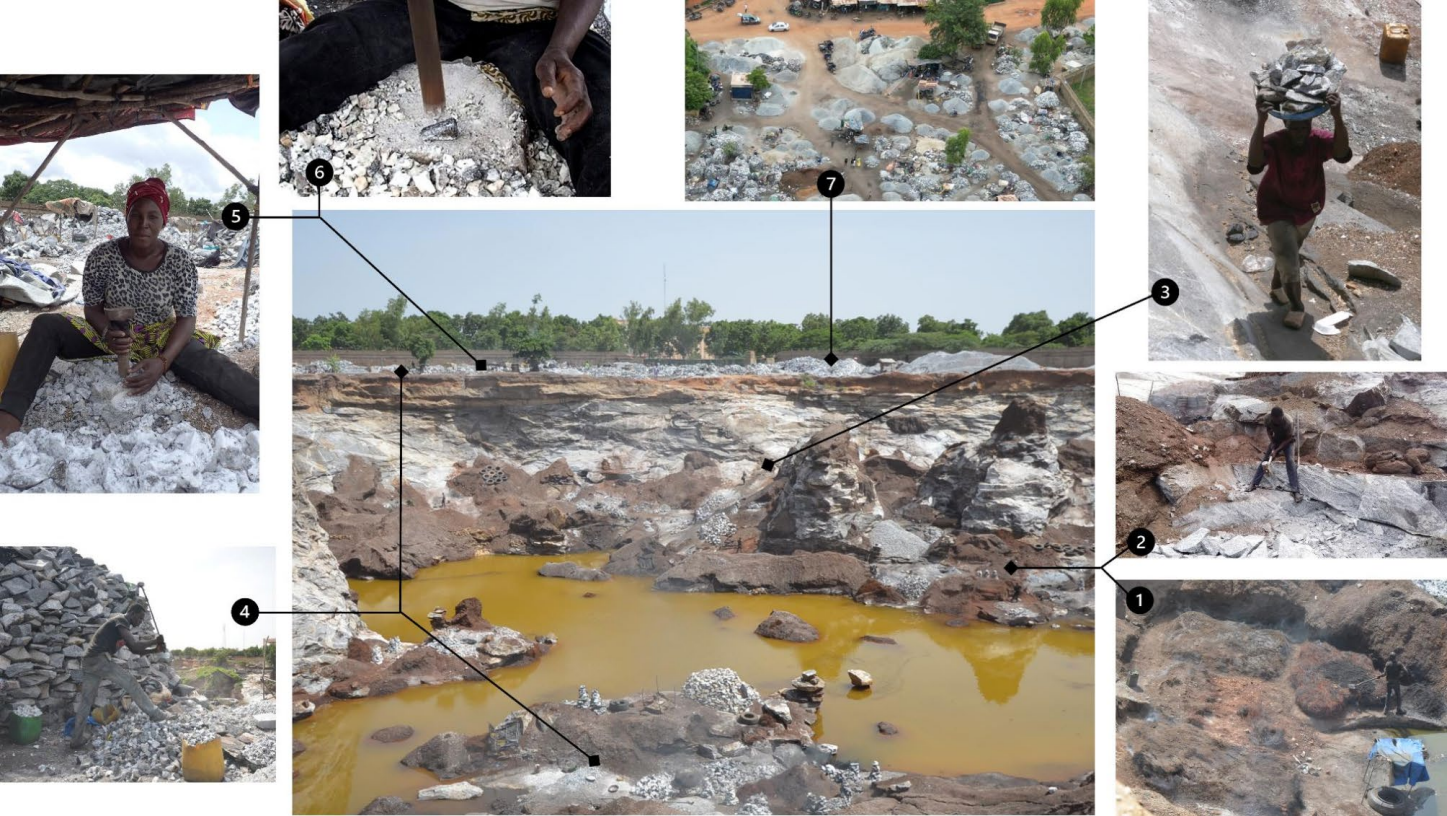
Process of artisanal granite mining in the Pissy quarry, Ouagadougou, Burkina Faso (August 2022): Burning tires create cracks in the rocks through thermal expansion (1). Manual cracking of the rocks into large pieces (2). Transport to the upper part of the mine (3). Reduction of rock size (4). Crushing rocks into small pieces (5) by using a metal bar (6). Stockpiling and sale (7).

**Fig. 7:**
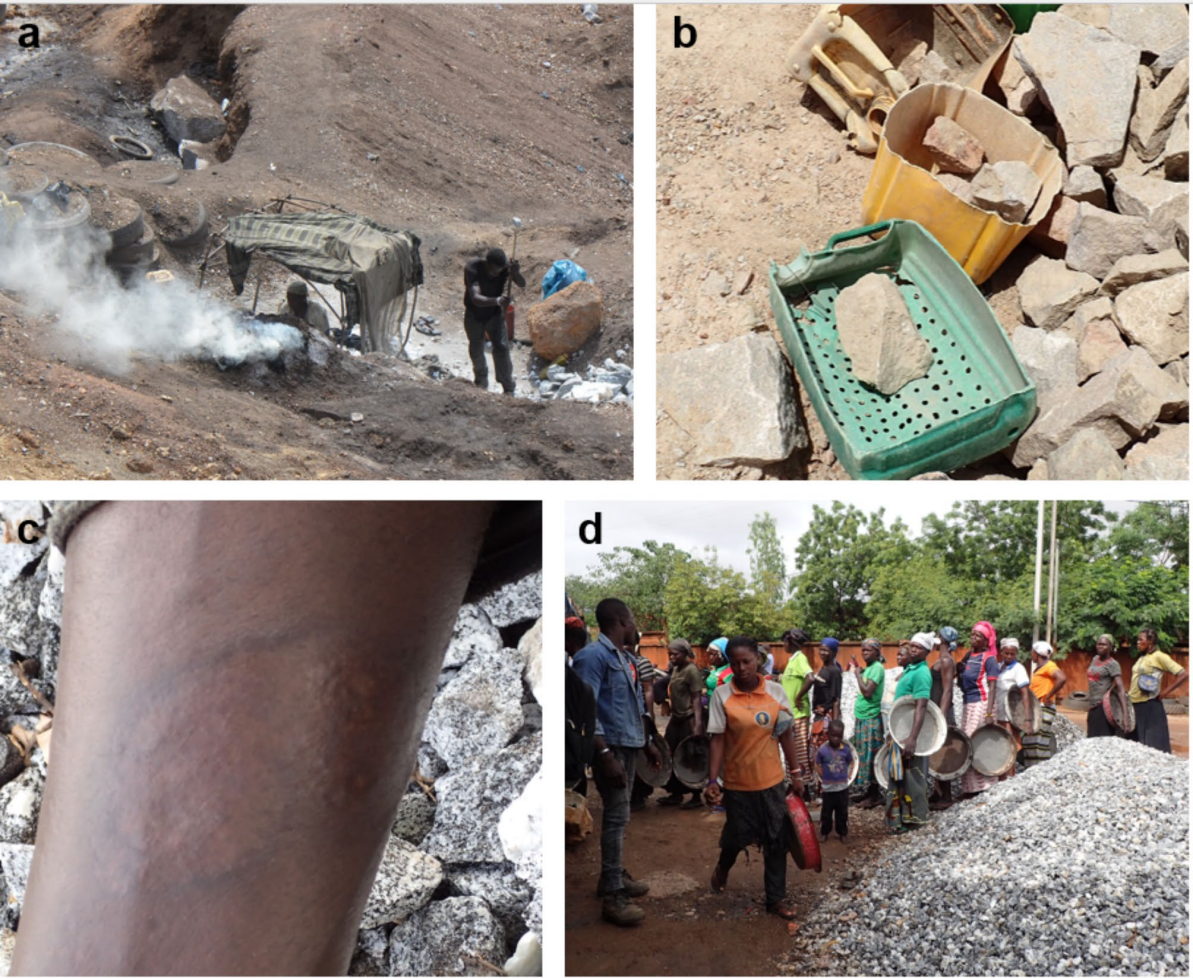
Informality of the artisanal granite mines of Pissy and Yagma, Ouagadougou, Burkina Faso (August 2022): (**a**) Simple, self-constructed workplace setups, (**b**) Basic tools used for sieving and transporting granite, (**c**) A scar on a worker’s forearm, indicating the hazardous working conditions and lack of protective gear, (**d**) Workers lining up to receive their daily cash payments for granite work.

The extraction process in Pissy and Yagma began with the controlled burning of tires covered with soil, a technique used to create cracks in the rocks through thermal expansion (Fig. 6 − 1). This step, which took between three and 15 days depending on the size of the rock and the number of tires used, was typically carried out by a smalle group of workers (*n* = 14 of the interviewees) who were also often responsible for the subsequent manual breaking of rocks into large pieces (Fig. 6 − 2). Thereafter, the larger rocks were transported to the upper part of the quarry, a task undertaken by a considerable number of interviewees (*n* = 31) using buckets, metal plates, or carts (Fig. 6 − 3). Rock size was reduced by manual hammering either before or after transportation to the upper part of the quarries by a small group of workers (*n* = 4, Fig. 6 − 4). The next step involved crushing the rocks into smaller pieces using a metal bar (Figs. 6 − 5 and 6–6). This task was the most labor-intensive and was carried out by the majority of the interviewed workers (*n* = 101). Following the crushing, a few workers (*n* = 4) were involved in sieving the material to separate the crushed rock from finer sand particles. Finally, the processed materials, ranging from manufactured sand over fine and coarse gravel to cobbles, were stockpiled for sale (Figs. 6 and 7). Only one interviewee reported being involved in the buying and selling process. Additionally, in Yagma, 12 interviewees were engaged in collecting sand on the surrounding terrain surface.

On average, the interviewees performed 1.5 extraction-related tasks, with 59% of them engaged in only one task and 41% involved in two to four different tasks along the value chain. The distribution of tasks varied significantly by gender (Chi Square Test: *p* = 1.26e-09): women were primarily engaged in crushing rocks, transporting rocks, and collecting sand, whereas men were more commonly involved in cracking rocks, making fires, and transporting rocks.

All operations at Pissy and Yagma were characterized by high levels of informality, as evidenced by several key factors. Firstly, there was a notable lack of regulatory compliance: those responsible for the Pissy quarry stated that the site had not been officially authorized for artisanal exploitation. Also, the representative from the DGC, the regulatory body for the extractive sector, noted that although permits are normally required, the regulations for artisanal mining have not yet been implemented. Nevertheless, 91% of the respondents did not perceive their activities as illegal, asserting that no unlawful extraction occurred. Meanwhile, 5% referenced specific regulations, such as limiting extraction to designated areas overseen by the military or prohibiting child labor on the site, as being potentially illegal. An additional 4% were uncertain about the legality of the activities. Regarding regulations, 85% of the respondents reported that no formal rules governed their work. Some exceptions were noted, such as prohibitions on fighting (5%) and other minor rules (7%). Additionally, 2% of respondents either did not know or did not provide an answer regarding the existence of regulations. Workers had complete freedom in choosing their workplace, as no formal permission was required to begin work (100% of respondents). However, in the lower part of Pissy, some individuals had established specific claims for burning and cracking rocks, requiring others to either seek permission or collaborate with them.

Secondly, the absence of worker contracts and legal protection was evident at Pissy and Yagma. The vast majority of interviewees worked alone, with 93% operating by themselves, 2% working in teams, and 5% experiencing both scenarios. Formal worker associations were largely absent, with only 2% of respondents reporting membership in an association. While there were efforts in Yagma to establish an association, such attempts were unsuccessful due to disagreements among workers about common objectives. In contrast, Pissy had an association known as “La Roche,” though participation remained minimal. The labour conditions at the sites reflected the lack of formal employment structures. Respondents reported working an average of 9.3 h per day and 6.2 days per week, highlighting the extensive working hours and the absence of social security benefits such as paid sick leave or vacation days. The informality of the artisanal granite quarries was further evidenced by the manual, small-scale nature of the operations. The workplaces were characterized by their simple setups, often relying on only very basic tools and equipment (Fig. 7a, b). Low initial investments facilitated low entry barriers into the industry. In Pissy, crushers typically spent around 2,210 FCFA (1,000 FCFA = 1.52 €) on basic tools, including a crushing iron and a tray. Further equipment such as hammers and shovels were mostly rented on-site on a daily basis (250–500 FCFA).

Hazardous working conditions were a prevalent concern at the artisanal granite quarries of Pissy and Yagma, largely due to the lack of safety gear. This led to a high incidence of injuries and accidents, which were identified as the main negative aspect of the job by 72% of the interviewees. Over three-quarters of the respondents (76%) reported having experienced injuries in the past. Among the injuries mentioned, 61 incidents (59% of all reported injuries) involved workers hitting their hands or feet with tools. Another 22 incidents (21%) were caused by splinters of rocks entering the skin or eyes, and 20 incidents (19%) involved various other types of injuries (Fig. 7c). Compounding the risk of injury was a lack of health insurance, which severely limited the workers’ access to proper medical treatment. Only 18% of those who were injured sought treatment at a hospital. The remaining 82% relied on traditional natural medicine to treat their injuries. Another indicator of the informality at the artisanal granite quarries was the exclusive reliance on cash-based transactions on all financial exchanges, including payments for labor and sale of materials (Fig. 7d).

Economic vulnerability and instability were significant challenges in the two artisanal granite quarries. In Pissy, the cost-revenue structure for workers, in particular those crushing rocks, revealed very low profitability (Table 1). Crushers had to account for material costs as well as for transportation (and an additional size reduction in Pissy) before processing it and selling the crushed rock. In Pissy, a crusher could produce around eight trays per day, while in Yagma, the crushed granite was sold in bulk or by bucket at a lower profitability.

**Table 1:** Cost-revenue structure for crushers at the artisanal granite quarries of pissy and Yagma, Ouagadougou, Burkina Faso.

	Category	Pissy (FCFA)	Yagma (FCFA)
Operational costs	Rock pile	1,000	2500
	Transport	500	500
	Size reduction	350	
Revenues	Revenue/unit	400/tray	300/bucket or 5000/pile
	Potential daily revenues	3350	900 (sold in buckets) or 1350 (sold in bulk)
Potential daily profits		735	280 (sold in buckets) or 400 (sold in bulk)

It should be noted that incomes for workers performing specific tasks were substantially higher; for example, those transporting rocks in Pissy generated an average of 3,550 FCFA per day. Nearly 98% of the respondents indicated that they had no other income. In line with this, 72% of the positive aspects about the work mentioned by the respondents referred to the ability to meet basic needs. Additionally, 14% of the responses highlighted that their work enabled them to provide education for their children. Other positive aspects (13%) included financing personal studies, avoiding idleness, and supporting family members. On the community level, the creation of jobs and income for the local population was mentioned by 26% of respondents, but was surpassed by access to building materials, cited as the main advantage by 60%. Another 8% were unsure of any positive effects, and 6% indicated there were none.

In addition to the already low incomes of crushers, market and seasonal fluctuations in sales and subsequent unstable incomes further aggravated their financial situation. Challenges differed significantly between the sites (Chi Square Test: *p* = 7.627e-06): In Pissy, 84% of respondents indicated that work became more difficult during the rainy season. The difficulties stemmed from the site partially filling with water, and the often torrential rainfalls, complicating the process of setting fires - requiring several dry days - and the transportation of materials due to the risk of slipping and falling rocks. Many workers turned to alternative livelihoods, such as farming and gold panning during the rainy season, leading to a reduced workforce and lower production volumes. In contrast, market conditions were better during this time, as demand increased due to increased building activities as rainwater could be used for construction. Consequently, granite was extracted and stocked during the dry season. In Yagma, the responses were more varied; 49% of respondents agreed that work during the rainy season was more difficult due to water filling the site, but 40% found the rainy season more favorable. They noted that rocks could be extracted from the surrounding areas of the quarry, which became inaccessible during the dry season when the soil was too hard to dig. Furthermore, the rain reduced the extraction-induced dust and enhanced sand availability. The remaining respondents were either indifferent to the seasonal variations (5%) or unsure (6%). Market downturns and fluctuations affected the operations and profitability of the granite quarries. These challenges led 58% of respondents to report difficulties in estimating daily revenues as they experienced periods of zero income lasting several days to weeks. Developments over time differed between the two sites (Chi Square Test: *p* < 2.2e-16). In Pissy, 93% of respondents indicated that conditions had been better in the past, with higher market demand leading to more regular sales, while the remaining 7% were unsure or noticed no change. In Yagma, however, only 12% agreed that conditions were previously better, while 79% of the respondents believed the situation had improved, despite worsened market conditions, due to increased sales prices in the area. Respondents further reported a recent increase in the number of laborers in the granite quarries, attributing this influx to (i) the perpetual insecurity in Burkina Faso that accelerates rural-urban migration as people seek safety in the country’s capital; (ii) relocations from other suburbs of Ouagadougou after a major flood in September 2009 devastated many parts of the city, as reported by workers in Yagma; and (iii) the COVID-19 pandemic that had led to the collapse of many small businesses since 2020, leaving a large number of individuals unemployed and turning to the extraction sector as a source of income. Together these factors boosted the workforce at the quarries, further intensifying competition for limited resources and opportunities.

The high level of informality surrounding operations at Pissy contributed to significant uncertainty about the workers’ future, exemplified by the sudden shutdowns of this mine in 2024. At the time of our survey, 37% of the respondents forecasted continued mining operations, while 27% indicated that the future fully depended on the landowners - namely the military in Pissy and the Alizeta company in Yagma. Another 19% were uncertain, attributing the decision to individual circumstances, while 17% believed operations would soon cease entirely. The outlook differed considerably between the sites: 87% of those optimistic about ongoing operations worked in Yagma, while 90% of the “no” responses and 64% of those attributing the future to landowners came from Pissy. The most common future aspiration (39% of the answers) was the desire to find a different job. This was followed by securing food (15%), and ensuring education, work, and well-being for their children (15%). Additionally, 9% of the responses highlighted the need for better housing, while 6% wished for peace. The remaining 17% of the responses were divided among transportation (5%), health, work materials (each 3%), and other miscellaneous aspirations (6%).

### Effects of artisanal granite extraction on air quality

The most commonly reported negative effect of granite quarrying on the community was the smoke generated from burning tires (48%). Additionally, 43% of the respondents stated they were unsure of the effects, and 8% reported no negative impact. There was a notable lack of awareness among respondents regarding the effects of granite extraction on the environment: 98% of the respondents were unaware about positive environmental effects whereas 2% stated there were none. Similarly, when discussing negative environmental effects, 98% were uncertain, while only 1% mentioned none and another 1% cited smoke pollution affecting air quality.

The air quality measurements conducted at the granite sites and control point showed stronger effects at Pissy than at Yagma (Table 2).

**Table 2:**
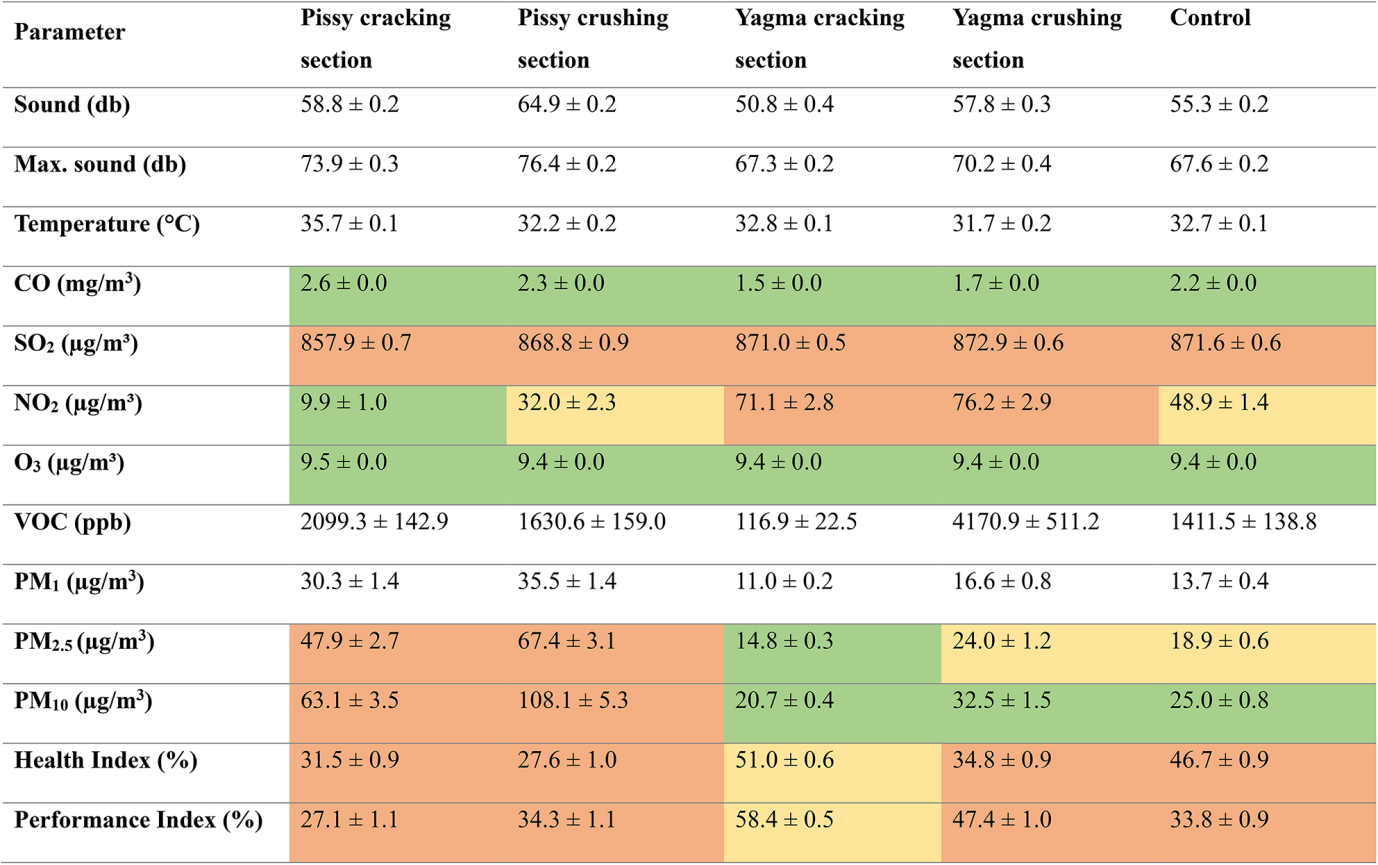
Average ± standard error of air quality parameters, Ouagadougou, Burkina Faso, 2023.

Parameters: Sound, max. sound (the highest sound level within a two-minute interval), temperature, carbon monoxide (CO), sulfur dioxide (SO₂), nitrogen dioxide (NO₂), ozone (O₃), volatile organic compounds (VOC), Particulate Matter (PM_1_, PM_2.5_, PM_10_), Health and Performance Indices (ranging from 0% (worst) to 100% (best), integrating several air quality parameters. Location: Study sites of Pissy and Yagma, each at the lower (cracking section) and upper parts (crushing section) of the granite mines; Control refers to a neutral control point within the city;. Color indicate comparison to World Health Organization (WHO) air quality guidelines^42^ for gaseous pollutants and PM (Green below WHO air quality guideline (AQG) level, orange: between AQG and the interim targets, red: above WHO interim target 1) and to air-Q (Corant GmbH, Leipzig, Germany) limits (green: above 70%, orange: between 50 and 70%, red: below 50%).

The Kruskal-Wallis Test revealed significant differences across all 13 parameters between the five locations, with p-values < 2.2e-16. Subsequently, Dunn’s Post-Hoc test showed that out of 130 pairwise comparisons (comparing each parameter across the four sites and control), 121 were significant. Notably, temperature showed the most exceptions with three comparisons being not significant, and there was one non-significant result each for maximum noise, SO₂, NO₂, O₃, performance, and PM_1_.

**Fig. 8:**
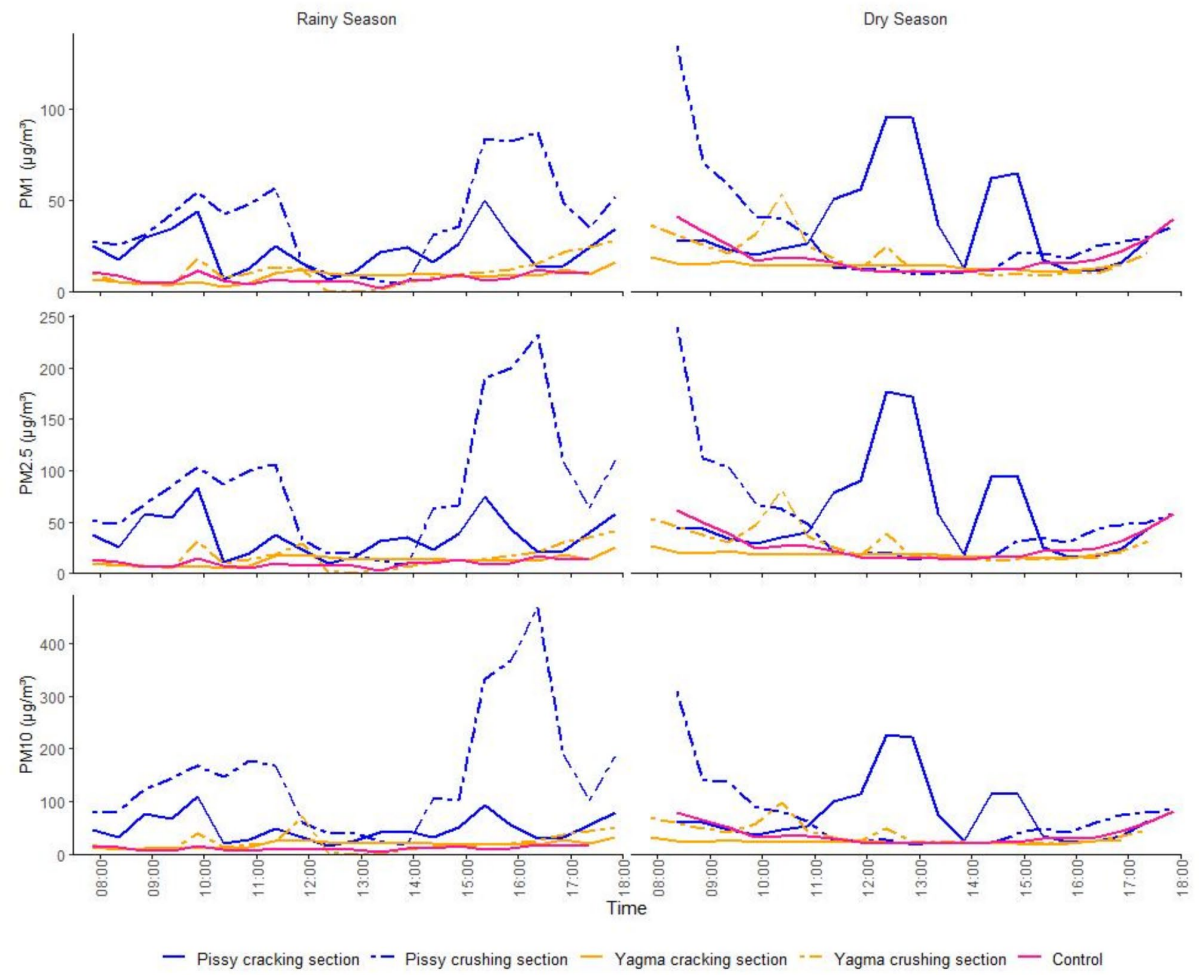
Distribution of particulate matter (PM) during the rainy season (September 2023) and dry season (October/November 2023), averaged over 30-minute intervals from 8 am to 5 pm, at the study locations and the neutral control point in Ouagadougou, Burkina Faso.

Distinct differences in PM levels were noted between the sites, leading to a detailed analysis of their development throughout the day. Yagma showed minimal peaks, while Pissy experienced pronounced peaks, potentially stemming from heavy burning events during these times (Fig. 8).

## Discussion

The inflow of sand to Ouagadougou from surrounding areas was smaller and from greater distances compared with the capital cities of Bamako (Mali) and Accra (Ghana). While our study identified sand extraction sites up to 165 km south of Ouagadougou, in Bamako and Accra, sand mining activities were limited to a radius of around 60 km from the city center. The estimated annual sand extraction volume of 1.8 million m^3^ (3.3 Mt) in Ouagadougou represents only 37% and 40% of the volumes in Bamako (4.86 million m^3^, 9.13 Mt) and Accra (4.55 million m^3^, 8.4 Mt), respectively^32,33^. The gap further widens on a per capita basis, with Ouagadougou requiring 2.5 kg of sand per person and day, compared with 8.9 kg in Bamako, 9 kg in Accra, and 18 kg globally^34–37^. This discrepancy is not due to lower demand for building materials, as Ouagadougou was projected to experience a population increase of 740,000 people between 2020 and 2025, compared with an increase of 563,000 inhabitants in Bamako and 274,000 in Accra, respectively. This corresponds to an urban growth rate of 4.7% in Ouagadougou, compared with 3.9% in Bamako and 2.1% in Accra^7^. Instead, it might be attributed to a more diverse supply of sand in Ouagadougou, which includes both small-scale mining of various sand sources within the city and crushed sand from artisanal granite quarries. Therefore, the truck counts from this study primarily reflect the rural-urban inflow of sand. The total demand for sand in the city of Ouagadougou may be slightly higher than reported, considering the additional contributions from inner-city mining activities. The lower quantities of sand compared with those of neighboring countries may also be attributed to the use of other building materials: The widespread use of locally extracted banco, or adobe, a mix of clay and renewable materials such as agricultural waste, is particularly notable in Ouagadougou’s poorer suburbs^11,38^.

Our findings of highly informal and hazardous working conditions corroborate with previous findings^25,26^, as well as with those of a stone quarry in Lubumbashi, Democratic Republic of Congo, where the lack of personal protective equipment was also noted^39^. The average daily incomes of crushers converted to average annual incomes of 236,000 FCFA in Pissy and 90,000 − 1128,000 FCFA in Yagma (average 6.2 workdays per week times 52 weeks). The income of the crushers in Pissy and in Yagma is thus below Burkina Faso’s national annual poverty line of 248,000 FCFA. The gap to the poverty line might even be bigger considering the mentioned seasonal and market fluctuations. This becomes particularly pronounced in the urban context, where only 16.6% of urban dwellers fall below the poverty line, highlighting the economic challenges faced by crushers in Pissy^40^. Our findings that refugees are part of the workforce of the granite quarries aligns with research by Soma^41^ on IDPs, which identifies Yagma as one of the receiving suburbs.

In neighboring Nigeria, several studies have been conducted to assess air quality parameters at quarries whereby results were similar to our measurements (Table 3).

**Table 3:** Average ± standard error of air quality parameter ranges of four studies in West-Africa.

Study	Madu et al.^43^	Bada et al.^44^	Okafor et al.^45^	This study
Sound (db)	79.13 ± 2.45–92.50 ± 2.55		38.99 ± 0.03–53.60 ± 0.01	50.8 ± 0.4–64.9 ± 0.2
Temperature (°C)	29.47 ± 1.05–33.90 ± 2.00			31.7 ± 0.2–35.7 ± 0.1
CO (mg/m^3^)	0.01 ± 0.01–1.17 ± 0.04	6.30 ± 0.57–6.87 ± 0.0	1.5 ± 0.0–3.95 ± 0	1.5 ± 0.0–2.6 ± 0.0
SO_2_ (µg/m^3^)	130 ± 100–800 ± 20	BDL	41.6 ± 2.8–58.3 ± 3.23	857.9 ± 0.7–872.9 ± 0.6
NO_2_ (µg/m^3^)	20 ± 10–220 ± 80		15.60 ± 1.37–49.87 ± 1.79	9.9 ± 1.0–76.2 ± 2.9
O_3_ (µg/m^3^)	20 ± 10–110 ± 20	BDL		9.4 ± 0.0–9.5 ± 0.0
PM_1_ (µg/m^3^)	1.22 ± 0.01–8.40 ± 0.21			11.0 ± 0.2–35.5 ± 1.4
PM_2.5_ (µg/m^3^)	1.83 ± 0.20–10 ± 0.32	65 ± 45–130 ± 10		14.8 ± 0.3–67.4 ± 3.1
PM_10_ (µg/m^3^)	2.17 ± 1.20–10.80 ± 1.21	74 ± 66–231 ± 18	360 ± 2.09–860.3 ± 1.59	20.7 ± 0.4–108.1 ± 5.3

Parameters: Sound, temperature, carbon monoxide (CO), sulfur dioxide (SO₂), nitrogen dioxide (NO₂), ozone (O₃), and Particulate Matter (PM_1_, PM_2.5_, PM_10_). Bada et al.^44^: Included drilling and crushing section, conversion of CO from ppm to mg/m^3^, BDL = Below detection limit. This study: In the listed ranges of values measurements from the control site are not included.

Obvious limitations of our air quality measurements were that: (i) Data collection was not conducted simultaneously at each site. Measurements taken on different days could thus suffer from fluctuating environmental conditions such as temperature. (ii) Lacking long-term measurements restricting the ability to assess temporal variations. (iii) Measurement points were central, potentially underrepresenting the exposure for workers closer to the source, particularly in the crushing section which were nearer to the louder noises produced by their tools repeatedly hammering on rocks. Future studies on the artisanal granite quarries should include exposure to crystalline silica dust to enhanceour understanding of its health implications.

We disagree with the recommendation of Kansole et al.^25^ to close down the Pissy quarry - based on concerns about environmental protection and workers’ health. Such closure may indeed reduce site-specific pollution, but it would boost environmental degradation elsewhere due to Ouagadougou’s growing demand for granite-based building materials. Additionally, such a closure would likely exacerbate the economic hardships of workers. Instead, we advocate for immediate improvements in working conditions, particularly through the enforced provision of appropriate protective gear and necessary training in its use as well as the investment in smaller tools like mobile hammer crushers. Over the long term, any decision to close the quarry should consider the needs for alternative income of the workers. Transition plans should comprise micro-credits and vocational training in new fields of work, ensuring a sustainable exit strategy from quarrying operations.

## Conclusions

Aggregate extraction around Ouagadougou is widespread encompassing various artisanal and mechanized sand and granite quarries. Despite the city’s rapid growth, the influx of sand from rural areas into the urban area remains relatively modest compared with similar inflows in neighboring capitals. The artisanal granite quarries of Pissy and Yagma, while crucial in providing construction materials to Ouagadougou, also contribute to environmental degradation, deteriorate air quality, and perpetuate precarious working conditions. Their consequences are especially pronounced among elderly women with limited educational backgrounds, who engage in rock crushing for meager and unstable incomes that fall below the national poverty line. Looking ahead, the workers’ futures remain dim. While rising urbanization will undoubtedly increase the demand for construction materials, political instability may trigger an even higher influx of workers displaced from other sectors or regions, leading to an even stronger competition for jobs at the quarries. Coupled with economic uncertainty, this may further destabilize demand and prices, intensifying the insecurities faced by workers in this volatile sector. This complex situation calls for informed decisions and subsequent law enforcement by policymakers to address the precarious health and social security conditions faced by workers while ensuring the long-term viability of urban construction.

## Electronic supplementary material

Below is the link to the electronic supplementary material.

Supplementary Material 1

## Data Availability

Data is provided within the manuscript or in supplementary information files.
