# Supplementary material for: Social-ecological dynamics of aggregate mining in Ouagadougou, Burkina Faso
Source: Sci Rep. 2025 Apr 28;15:14811. doi: 10.1038/s41598-025-98929-6 (PMC12037907; doi:10.1038/s41598-025-98929-6)
Supplement: Supplementary file 1 — Supplementary Material 1 [file 41598_2025_98929_MOESM1_ESM.docx]

**Electronic supplementary material**

**S1 Data.** Overview of survey questions and response data of workers of the Pissy and Yagma granite mines, Ouagadougou, Burkina Faso. <https://doi.org/10.6084/m9.figshare.28029041>

**S2 Data.** Air quality data of the Pissy and Yagma granite mines, Ouagadougou, Burkina Faso. <https://doi.org/10.6084/m9.figshare.28029074>

**S3 Data.** Truck count data and calculations of loaded sand trucks entering Ouagadougou, Burkina Faso. <https://doi.org/10.6084/m9.figshare.28029086>
